# Spine surgery outcome in patients who sought compensation after a motor vehicle accident: a retrospective cohort study

**DOI:** 10.1186/s12893-016-0192-8

**Published:** 2016-11-21

**Authors:** Pooria Sarrami, Rafael Ekmejian, Justine M. Naylor, Joseph Descallar, Robindro Chatterji, Ian A. Harris

**Affiliations:** 1 South Western Sydney Clinical School, UNSW, Sydney, Australia; 2 Institute of Trauma and Injury Management, Agency for Clinical Innovation, Level 4, Sage Building, 67 Albert Avenue, Chatswood, Sydney, NSW 2067 Australia; 3 South Western Sydney Local Health District, Liverpool Hospital, Liverpool, Australia; 4 Ingham Institute for Applied Medical Research, Sydney, Australia

**Keywords:** Spine surgery, Decompression, Clinical outcome, Road traffic accidents, Compensation

## Abstract

**Background:**

Back and neck pain are common after road traffic injury and are treated by spine surgery in some cases. This study aimed to describe the outcomes of spine surgery in people who made an insurance claim after road traffic accidents without an associated spinal fracture or dislocation.

**Methods:**

This study was a retrospective cohort based on insurers’ data of Compulsory Third Party (CTP) claims. File audit and data extraction were undertaken using a study-specific proforma. Primary outcomes were ongoing pain and symptoms, complications, return to work and pre-injury duties, and ongoing treatment 2 years following spine surgery. Secondary outcomes were health care costs based on data provided by the insurers.

**Results:**

After screening 766 files, 90 cases were included (female: 48; mean age: 46 years). Among the subjects who were working prior the injury, the rate of return to work was 37% and return to pre-injury duties was 23% 2 years following the surgery. The average number of appointments with health care professionals in the 1 year after surgery was 21, compared to 10 for the 1 year prior to surgery (*p* = 0.03). At 2 years following the initial surgery, 21% of claimants had undergone revision spine surgery; 68% reported ongoing back pain and 41% had ongoing radicular symptoms. The difference between costs 1 year before and after surgery (excluding surgical costs) was statistically significant (*p* = 0.04). Fusions surgery was associated with higher total costs than decompression alone. After adjusting for surgery type, lumbar surgery was associated with higher costs in the 1 year after surgery and total surgical costs compared to cervical surgery.

**Conclusions:**

The majority of claimants continued having clinical symptoms, continued using health care and did not return to work despite undertaking spine surgery.

**Electronic supplementary material:**

The online version of this article (doi:10.1186/s12893-016-0192-8) contains supplementary material, which is available to authorized users.

## Background

Road traffic crash casualties are estimated to cost $17b in Australia each year, with New South Wales (NSW) having the highest total cost compared to other states [1]. Among the annual cost of road traffic crash casualties, 56% are related to human costs, including: medical treatment and rehabilitation; long-term care; labour in the workplace and quality of life [1].

Back and neck pain are common after road traffic injury [2]. Recovery of back pain is lengthy and the median time to claim closure is reported to be 505 days [2]. Post-accident neck and back pain are also predictors of chronicity in whiplash after motor vehicle crashes [3–5]. A considerable number of patients who are involved in a road traffic accident will pursue a compensation claim, mostly through a Compulsory Third Party (CTP) scheme. In NSW, CTP insurance covers all persons involved in a motor vehicle crash on public roads who are not at fault. Surgery is a one of the treatments used for back pain [6–8] including following motor vehicle injury and surgery costs can be covered by the CTP scheme for such claimants.

Emerging evidence suggests seeking compensation can be associated with poorer health outcomes [9]. A previous study retrospectively reviewed outcomes of spine surgery for patients without fracture or dislocation in an Australian worker’s compensation cohort. Data were collected from WorkCover NSW and insurer agents and it was found 77% of patients needed ongoing treatment 2 years post spine surgery [6]. However, there have been no reports on the outcome of patients who undergo spine surgery under CTP scheme after road traffic accidents. Therefore, this study aimed to explore the outcomes of such patients. Specifically, we aimed to determine rates of ongoing pain and symptoms, complications and ongoing treatment and health care use, and to determine predicting capability of age, gender, surgery type, surgery location, and the socio-economic status of residential area.

## Methods

We studied a retrospective cohort of claimants identified using information from three insurers, covering 70% of the CTP insurance market in the study location [10]. Claimants were 18 years or older and had undergone spinal surgery after a road collision during 2005–2011 and were treated under CTP insurance. Those with acute fracture-dislocations were excluded, as these conditions represent a different population to those treated for ongoing, chronic pain after injury [6]. File audit and data extraction were undertaken by three researchers using a study-specific proforma (See Additional file 1).

Primary outcomes were ongoing pain and symptoms, opioid consumption, revision surgery, return to work (RTW), return to pre-injury duties (PID) 2 years following spine surgery. In addition, healthcare use (e.g. physiotherapy, pain management, hydrotherapy) was measured and compared 1 year before and after surgery. Secondary outcomes were health care costs (total health care costs insurers spent on the subjects and costs 1 year before surgery and 1 year after surgery) based on data provided by the insurers.

Insurers’ data systems varied; one relied on paper files and Australian Medical Association (AMA) codes and two relied on electronic files and Abbreviated Injury Scale (AIS) codes. Therefore, strategies to identify cases were different for each insurer. To identify cases with spine surgery, two comprehensive lists of AMA and AIS codes were used (See Appendix). Presence of a surgical procedure could not be determined via automated systems and was done manually by screening patients’ files. However, the process of screening was facilitated based on the dates of significant payments (more than $2000) as it was assumed that spinal surgery in Australia could not be performed for less than $2000. Claimants’ files, including reports of imaging studies, were examined and cases were excluded if there was any indication of fractures or dislocations. Socio-demographic data (age, gender, and postcode), car collision history (position at the time of car collision and seat belt use), data related to surgery (surgery type and location), health care use and RTW status were gathered from the files.

Claimants studied in this research could have undertaken spine surgery only if insurers’ medical assessments indicated their back pain and symptoms are mainly attributable to the recent car accident rather than any prior spine issues. For this reason, claimants’ past history was not included in data analysis.

Rates of ongoing back pain and ongoing radicular symptoms, obtained from claimants’ files, during 2 years following surgery were recorded. In addition, the rates of opioid consumption before and after surgery were recorded. McNemar test was used to compare the rate of opioid consumption in claimants before and after surgery.

Rate of return to work (RTW) and return to pre-injury duties (PID) during 2 years after surgery were explored among the subjects who were working either part-time or full time before the accident. RTW indicated that subjects were working in any capacity after surgery, while PID indicated that subjects were working at the same level (part-time or full-time) as they did before the accident. These two rates were recorded as binary variables.

In order to explore health care use, the number of appointments that the subjects had with health care professionals for services such as physiotherapy, pain clinic, hydrotherapy and psychotherapy in the 1 year prior to the surgery were considered and was compared with the number of appointments in the same claimants 1 year after surgery using paired *t*-test it. Only those participants who had 1 year or more between the car accident and the surgery were included for that part of the analysis compared before and after surgery. All participants had data related to at least 2 years after surgery. In addition, the rate of revision surgery 2 years after the initial surgery was recorded. Revision surgery was defined as repeat surgery (of any type) to the same spine region of the initial surgery (cervical or lumbar) for ongoing symptoms (and not for any new incidence or pathology).

Data related to health care costs included total health care costs that insurers paid for each claimant and costs insurers paid in the 1 year before and after surgery. Total health care costs included all payments made by the insurers for treatment of each subject, including outpatient services, consultations fees, medication costs, hospital fees, surgeon fees, implant costs and inpatient costs. Total health care costs did not include non-health related payments. Costs 1 year before surgery and 1 year after surgery did not include costs directly related to surgery (surgeon fees, inpatient costs, implant costs and hospital costs). Acute health care costs at public hospitals are covered by the NSW Motor Accidents Authority (MAA) and are not included in the health care costs before surgery. Costs in the 1 year before surgery were compared with costs in the same claimants in 1 year after surgery using paired *t*-test.

We analysed whether age, gender, surgery type, surgery location and the Socio-Economic Indexes for Areas (SEIFA; calculated based on subjects’ residential postcode) were associated with each of the outcomes. Surgery type was considered as either fusion (with or without decompression) or decompression alone. Univariate logistic regression was used to determine if there were associations between the predictors and binary categorical outcomes (improved back pain, work status, revision surgery, and opioid use after surgery). Negative binomial regression was used to analyse the number of times health care was used after surgery as a count. Data analyses were performed using SAS 6.1 (Cary, NC, USA).

## Results

**Fig. 1:**
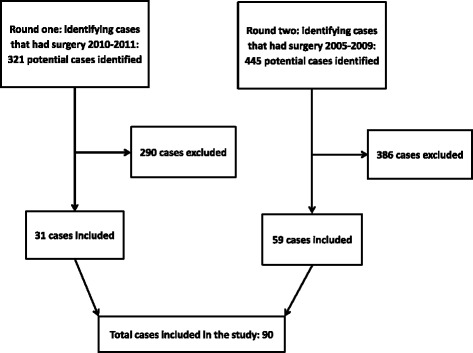
Flowchart of screening and inclusion process

In the first round of data collection, three insurers provided access to 321 cases that potentially had spine surgery during 2010–11. After screening 321 files from the three insurers, 31 cases were included. As the number of the included claimants was low, an ethics amendment was obtained and the study inclusion period was extended to include subjects having surgery during 2005 to 2011. One of the insurers did not continue its collaboration with the study due to their resource limitations. A further 445 cases from the remaining two insurers were screened and 59 cases were included from the other two insurers. The reasons for exclusion were: not identifying any record of spine surgery (82%), spine fracture (15%), surgery performed outside of designated time period of this study (4%) and age under 18 (1%). Figure 1 illustrates the overall process of screening and inclusion. The exclusion was only due to ineligibility.

Of the 90 included claimants, 48 were female (53%). The mean age at the time of claim was 46 years (SD: 11.9, range: 23 to 73). The majority of subjects were the driver of their car at the time of accident (68%). The rest were passengers (22%), motorcyclists (6%) or pedestrians (3%) (Table 1).

**Table 1:** Demographic information of the claimants included in the study

	Position of claimant at the time of accident							
	Driver		Passenger		Pedestrian		Motorcyclist	
	Male	Female	Male	Female	Male	Female	Male	Female
Age less than 35	4	9	1	3	0	0	1	0
Age between 36 and 50	9	16	4	4	2	0	3	0
Age between 51 and 65	13	6	2	4	0	0	0	1
Age more than 66	2	1	0	1	0	1	0	0

For those who could use a seat belt, 96% claimed they used it at the time of accident. Included claimants were from wide range socioeconomic statuses based on the postcode of their living place.

Decompression was the most common type of surgery performed (56%), followed by the combination of fusion and decompression (34%) and fusion alone (10%). Approximately half of the procedures were undertaken on the lumbar spine (52%), the rest were on the cervical spine. The surgery levels ranked from the highest frequency were: C5/6 (22%), L4/5 (21%), multiple levels (20%), L5/S1 (19%), C6/7 (13%) and other single levels (4%). The mean time between accident and spine surgery was 386 days (SD: 271, range: 41 to 1552).

Insurer one provided health care costs data of 41 cases out of 42 cases; however, insurer two provided cost data for only 16 cases (of 42 cases), because the insurer paid a lump sum for the remaining participants and could not separate health care costs. Insurer three, who did not participate in round 2, provided access to cost data of all 6 cases. The average total health care cost per patient was $59,145 (SD: 35,817; range: $11,064 to $164,189). The total health care cost by surgery type was: decompression surgery $47,875; fusion surgery $60,130 and decompression and fusion together $75,016.

### Outcome of claimants after spine surgery

In 68% of claimants, there were reports of ongoing back pain 2 years following surgery. Similarly, 41% of participants had ongoing radicular symptoms following surgery. In addition, opioid consumption did not statistically change after surgery (Table 2). Opioid use was not able to be determined in all claimants. The missing data indicates we have identified the minimum rate of opioid use. Before surgery 48% (23/48) of participants used opioids and after surgery this figure was 57% (39/68). Statistically these was no significant difference between these rates (McNemar test, *p* = 0.80, Table 2).

**Table 2:** Cross-tabulation of opioid use before and after surgery

		Opioid use after surgery		
		Yes	No	Total
Opioid use before surgery	Yes	15	7	22
	No	9	14	23
	Total	24	21	45

Before the accident, 78% of the claimants were working either full-time or part-time. This rate was reduced to 37% after the accident and did not change after surgery (Table 3). Among the subjects who were working either part-time or full-time prior the injury, the rate of RTW was 37% and return to PID was 23% 2 years following the surgery.

**Table 3:** Comparison of claimants’ status before and after surgery

Variable	Number of claimants with data	Pre-injury	Pre-surgery	Post-surgery
Percentage of claimants working either full-time or part-time	Pre-injury: 88 Pre-surgery: 84 Pre-surgery: 83	78%	37%	37%
Average health care costs during one year	Before and after surgery: 29	-	$2289	$4271
Average number of appointments with health services during one year	Before and after surgery: 23	-	10	21
Percentage of claimants using opioids	Before surgery: 48 After surgery: 68	-	48%	57%

Claimants had on average 10 appointments with health care professionals for services such as physiotherapy, pain clinic, hydrotherapy and psychotherapy in the 1 year prior to the surgery, while they averaged 21 appointments in the 1 year after surgery (Tables 3 and 4). This difference is statistically significant (paired sample *t*-test, *p* = 0.03). During the 2 years following the initial surgery, 21% of patients had undergone revision surgery.

**Table 4:** Comparison of claimants’ health care use 1 year before and after surgery

	Average number of appointments in claimants before surgery	Average number of appointments in claimants after surgery
Psychology	3	7
Physiotherapy	8	16
Pain management	2	0
Massage therapy	0	7
Hydrotherapy	2	13
Psychiatrist	1	3
Occupational therapy	0	1
Social work	2	2
Rehabilitation	0	2
Chiropractic	2	1

Average health care costs that the insurers paid for claimants in the 1 year before surgery was $2289, while this figure during the 1 year after surgery was $4.271 (Table 3). The difference between before and after surgery health care costs was statistically significant (paired sample *t*-test, *p* = 0.04). The calculated costs before and after surgery did not include costs directly related to surgery, such as surgeon fees, implant fees or hospital costs.

There were no significant associations between the potential predictors and work capacity, health care use, ongoing pain, rates of revision surgery or opioid use.

There were no significant associations between age, gender, SEIFA deciles, or surgery type and costs 1 year after surgery. However, after adjusting for surgery type, surgery location (lumbar versus cervical spine) was found to be a predictor of costs after surgery (*p* = 0.04). The average costs during 1 year after surgery for claimants with lumbar surgery was $8784, while for cervical surgery it was $4201.

There were no significant associations between age, gender and SEIFA deciles with total costs. However, surgery type was a predictor of total costs (*p* = 0.02; average total cost for decompression: $50,550 and for fusion with and without decompression: $71,543). Surgery location was also identified as a predictor of total costs (*p* = 0.02; average total cost for lumbar surgery: $68,030; and for cervical surgery: $55,482).

## Discussion

In this study, undertaking spine surgery under CTP compensation after motor vehicle trauma, compared to pre-surgery, was associated with increased health care use, high rates of ongoing pain and low rates of return to pre injury duties at 2 years post-surgery, and increased non-surgical health care costs 1 year after surgery. The rate of revision surgery observed here (21%) was in the range of previous studies that reported 9.2 to 27% [6, 11, 12]. The rate of RTW was 43%, which is similar to other studies that have reported 26 to 50% RTW [6, 12].

These results are similar to several other studies that reported poor outcomes of spine surgery under workers’ compensation for spine conditions without fracture or dislocation [6, 11, 12]. Studies undertaken on workers’ compensation cohorts have been criticised for considering a specific population with particular types of occupation [13]; however, this criticism is not applicable to this study as claimants did not belong to a particular working group. Results of this study are similar to previous studies indicating that spine surgery does not reliably improve RTW or reduce pain or health care use in patients with no fracture or dislocation.

It is notable that patients can have more routine appointments with allied health services post-operatively with the aim of achieving a better outcome, which does not necessarily reflect failure or inadequacy of surgical treatment. Nevertheless this increased health care use indicates that the cost of surgical therapies for claimants and/or insurers is not limited to the direct costs.

In addition, while spine surgery is a costly procedure, it also led to a further increase in claimants’ health care costs after surgery due to further use of health services such as physiotherapy. The lack of improvement may be due to the complex nature of chronic pain which is related to physiological, psychological and social factors [14].

The decision for surgery should be based on shared decision making with patients that includes providing information on surgery outcomes. Therefore, reliable information is needed regarding the outcomes of surgery.

The retrospective research methodology employed in this study had the advantage of ease of access to available data in a relatively short-time, however, this method imposed limitations. Insurers could not provide cost data related to 27 cases. In addition, files did not include the needed information in some cases, for example 17 files did not have adequate data on work status of claimants. These missing data were random and we do not have any reason to assume the missing values would be systematically different from the available data. For each spine surgery outcome, the patient population have varying sample sizes since this information was not available in every patient (Table 3). This would lead to reduced statistical power when analysing the association between predictors and outcomes. In addition, relying on the insurance data for, we have not access to potential health care use and costs that claimants may have had out of the insurance system. Due to our reliance on medical notes submitted to insurers, it is likely that we have underestimated the complications, such as revision surgery, as these may have occurred outside the compensation system.

A lack of control group is another limitation of retrospective studies like this. While we have observed a lack of improvement in the claimants we cannot ascertain the outcome of those who did not undergo surgery. It is also notable that we could not use standardised tools to measure the quantity of pain before and after surgery; we could only compare existence of pain based on the contemporaneous medical notes reported whether the patients felt better, worse or not changed. In addition, we did not have access to claimants’ history before the injury, including use of opioids, which may influence some of the outcomes measured.

Despite the limitation of the method of retrospective inspection of claimants’ files, insurers can be a valuable source of data for similar studies. However, in order to make recommendations and address the existing uncertainties on the application of spinal surgery for claimants who have pain following a motor vehicle accident, there is a need for more rigorous methods such as randomised control trials, [15] prospective cohort studies or registries.

Future studies should utilise prospective research methods and include additional dimensions such as patients’ expectation of surgery [16] and psychological predictors [17]. As previous studies have reported that the outcome of spine surgery varies between diagnostic subgroups [18], outcomes of different groups of patients should be compared in those with and without using compensation. In addition, patient reported outcomes of revision surgeries could also be investigated.

## Conclusion

This study does not support the use of spine surgery under CTP compensation for claimants without a fracture or dislocation. Comparative studies are required to determine the relative effectiveness of surgery in this environment. Until such data are available, based on the high costs of surgery and the limited benefits for this particular patient group, utilisation of other pathways of care is suggested.

## Appendix

### Injury codes used to identify potential cases with spine surgery

#### AMA (Australian Medical Association) codes

MT030, MT050, MT055, MT060, MT070, MT080, MT090, MT100, MT110, MT130, MT140, MT145, MT150, MT160, MT170, MT180, MT190, MT200, MT210, MT220, MT230, MT240, MT250, MT260, MT270, MT280, MT290, MT300, MT310, MT320, MT330, MT340, LT045, LT055, LT075, LT135, LT145, LT155, LT165, LT175, LT185, LT195, LT205, LT215.

#### AIS (Abbreviated Injury Scale) codes

Cervical spine: 650299.2, 650200.2, 651202.2, 650203.3, 650205.3, 630260.2, 640278.1.

Thoracic spine: 650499.2, 650400.2, 650402.2, 650403.3, 650405.3, 630499.2, 640478.4.

Lumbar spine: 650699.2, 650600.2, 650602.2, 650603.3, 650605.3, 640678.1.

Exacerbation: Z878.

## Additional file

Additional file 1: Study-specific proforma. (DOCX 51 kb)

## Abbreviations

AIS: Abbreviated injury scale
AMA: Australian Medical Association
CTP: Compulsory Third Party
MAA: Motor Accidents Authority
NSW: New South Wales
PID: Pre-injury duties
RTW: Return to work
SEIFA: Socio-Economic Indexes for Areas
