# Supplementary material for: Spine surgery outcome in patients who sought compensation after a motor vehicle accident: a retrospective cohort study
Source: BMC Surg. 2016 Nov 21;16:76. doi: 10.1186/s12893-016-0192-8 (PMC5116816; doi:10.1186/s12893-016-0192-8)
Supplement: Additional file 1: — Study-specific proforma. (DOCX 51 kb) [file 12893_2016_192_MOESM1_ESM.docx]

**Demographic information**

| **Gender:** | ☐ Female ☐ Male | **Patient’s postcode:** |  |
| --- | --- | --- | --- |
| **Date of birth:** | DD/MM/YYYY | **Age (at the time of claim):** | DD/MM/YYYY |
| **Occupation:** |  | | |
| **Pre-injury work status:** | ☐ Full time work or education ☐ part-time ☐ casual ☐ no work | | |
| **Post-injury (pre-operation) work status:** | ☐ Full time work or education ☐ part-time ☐ casual ☐ no work | | |
| **Post-operation work status:** | ☐ Full time work or education ☐ Part-time ☐ Casual ☐ No work | | |

**Medical History**

| **Is there any record that the patient had a history of spine problems before injury?** | |
| --- | --- |
| ☐ No | ☐ Yes (please specify) |

**Health care usage pre-surgery**

| **Medicine**: | Name:  Dosage per day: | Name:  Dosage per day | Name:  Dosage per day: |
| --- | --- | --- | --- |
|  | Name:  Dosage per day | Name:  Dosage per day | Name:  Dosage per day |
| **Psychological services:** | Number of sessions: from: DD/MM/YYYY to: DD/MM/YYYY | | |
| **Physiotherapy:** | Number of sessions: from: DD/MM/YYYY to: DD/MM/YYYY | | |
| **Pain management:** | Number of sessions: from: DD/MM/YYYY to: DD/MM/YYYY | | |
| **Acupuncture:** | Number of sessions: from: DD/MM/YYYY to: DD/MM/YYYY | | |
| **Massage :** | Number of sessions: from: DD/MM/YYYY to: DD/MM/YYYY | | |
| **Hydrotherapy:** | Number of sessions: from: DD/MM/YYYY to: DD/MM/YYYY | | |
| **Others (specify):** | Number of sessions: from: DD/MM/YYYY to: DD/MM/YYYY | | |

**Car collision history**

| **Date of car collision:** | DD/MM/YYYY |
| --- | --- |
| **Position at the time of car collision:** | ☐ Driver ☐ Passenger ☐ Pedestrian |
| **Seat belt used during the car collision:** | ☐ Yes ☐ No ☐ Data not available |

**Surgery details**

| **Date of admission:** | DD/MM/YYYY | **Date of operation:** | DD/MM/YYYY |
| --- | --- | --- | --- |
| **Primary diagnosis:** |  | | |
| **Revision diagnosis:** |  | | |
| **Surgery type:** | | | |
| ☐ Decompression (☐ Laminectomy and/or ☐ Discectomy) | | | |
| ☐ Disc replacement | | | |
| ☐ Fusion | | | |
| **Complications :** | | | |
| ☐ None | | | |
| ☐ Readmission (specify):  ☐ Reoperation (specify):  ☐ VTE  ☐Others (specify): | | | |
|  | | | |
|  | | | |
|  | | | |

**Health care usage 24 months after surgery**

| **Medicine**: | Name:  Dosage per day | Name:  Dosage per day: | Name:  Dosage per day: |
| --- | --- | --- | --- |
|  | Name:  Dosage per day | Name:  Dosage per day | Name:  Dosage per day |
| **Psychological services:** | Number of sessions: from: DD/MM/YYYY to: DD/MM/YYYY | | |
| **Physiotherapy:** | Number of sessions: from: DD/MM/YYYY to: DD/MM/YYYY | | |
| **Pain management:** | Number of sessions: from: DD/MM/YYYY to: DD/MM/YYYY | | |
| **Massage:** | Number of sessions: from: DD/MM/YYYY to: DD/MM/YYYY | | |
| **Hydrotherapy:** | Number of sessions: from: DD/MM/YYYY to: DD/MM/YYYY | | |
| **Acupuncture:** | Number of sessions: from: DD/MM/YYYY to: DD/MM/YYYY | | |
| **Others (specify):** | Number of sessions: from: DD/MM/YYYY to: DD/MM/YYYY | | |

| **Date data collection completed:** | DD/MM/YYYY |
| --- | --- |
| **Person completing data collection:** |  |
